# Matrix Metalloproteinase-dependent turnover of cartilage, synovial membrane, and connective tissue is elevated in rats with collagen induced arthritis

**DOI:** 10.1186/1479-5876-10-195

**Published:** 2012-09-20

**Authors:** Anne Sofie Siebuhr, Jianxia Wang, Morten Karsdal, Anne-C Bay-Jensen, Jin Y, Zheng Q

**Affiliations:** 1 Nordic Bioscience, Herlev, Denmark; 2 Nordic Bioscience, Beijing, China; 3 Nordic Bioscience, Beijing, China; 4 Nordic Bioscience, Beijing, China

**Keywords:** Collagen balance, Rheumatoid arthritis, Matrix metalloproteinase, Synovial membrane, Cartilage, Connective tissue

## Abstract

**Background:**

Rheumatoid arthritis is a disease affecting the extracellular matrix of especially synovial joints. The thickness of the synovial membrane increases and surrounding tissue degrades, leading to altered collagen balance in the tissues. In this study, we investigated the altered tissue balance of cartilage, synovial membrane, and connective tissue in collagen induced arthritis (CIA) in rats.

**Methods:**

Six newly developed ELISAs quantifying MMP-derived collagen degradation (C1M, C2M, and C3M) and formation (P1NP, P2NP, and P3NP) was used to detect cartilage turnover in rats with CIA. Moreover, CTX-II was used to detect alternative type II collagen degradation and as control of the model. 10 Lewis rats were injected with porcrine type II collagen twice with a 7 day interval and 10 rats was injected with 0.05 M acetic acid as control. The experiment ran for 26 days.

**Results:**

A significant increase in the degradation of type I, II, and III collagen (C1M, C2M, and C3M, respectively) was detected on day 22 (P = 0.0068, P = 0.0068, P < 0.0001, respectively), whereas no significant difference in formation (P1NP, P2NP, and P3NP) was detected at any time point (P=0.22, P=0.53, P=0.53, respectively). The CTX-II level increased strongly from disease onset and onwards.

**Conclusion:**

A nearly total separation between diseased and control animals was detected with C3M, making it a good diagnostic marker. The balance of type I, II, and III collagen was significantly altered with CIA in rats, with favour of degradation of the investigated collagens. This indicates unbalanced turnover of the surrounding tissues of the synovial joints, leading to increased pain and degeneration of the synovial joints.

## Background

Rheumatoid arthritis (RA) is a chronic inflammatory disease, which primarily affects the extra-cellular matrix (ECM) of the synovial joints [1]. The exact etiology of the disease is still unknown, but several factors such as gender [2], genetics [3,4] and antigens [1] are thought to be involved. The extensive inflammation of the synovial membrane (i.e. synovitis) and other joint tissues induces secretion of proteolytic enzymes leading to degradation of cartilage and elevated bone turnover [5-7].

The synovial membrane is divided into two separate layers, an intimate layer of cells embedded in ECM and a sub-intimae layer of loose connective tissue [8,9]. The first layer consists of two types of cells, fibroblast-like synoviocytes and macrophages. These cells form a capsule enclosing the joint, containing type I and III collagen [9,10]. In healthy individuals this layers is approximately 1–4 cells thick, whereas it can be up to 12 cells thick in individuals with RA [1,8]. The increased amount of cells results in increased concentrations of degradative enzymes, such as matrix metalloproteinases (MMP) and aggrecanases, which are released into the synovial fluid, leading to increased degradation and formation of the surrounding ECM of cartilage, connective tissue, and the synovial membrane itself [11-13]. It is well known that MMP-1, -3, -9, and −13 is upregulated with RA [13-15]. These MMP’s are important proteolytic enzymes in ECM breakdown as MMP-1 degrades type I, II and III collagen, MMP-3 degrades, amongst other ECM proteins, type III collagen and the proteoglycans of cartilage, MMP-9 degrades type IV and V collagens, and MMP-13 degrades type I, II, and III collagen, however type II collagen more efficiently than type I and III collagen.

The protein fragments derived from the proteolytic enzyme cleavage, called neo-epitopes, are potentially released from the tissues and may enter into the circulation and thereby serve as biochemical markers indicating the degree of tissue destruction. There are already several commercial biochemical markers availably utilizing neo-epitopes; CTX-I [16], PIIANP [17], COMP [18], and ICTP [19].

In this study we used newly developed biochemical markers based on neo-epitopes of type I, II, and III collagen degradation (C1M [20], C2M [21], C3M [22]) and formation (P1NP [23], P2NP(unpublished), P3NP(unpublished)) to describe the turnover of cartilage, synovial membrane, and connective tissue. The degradation markers are all derived from cleavage with RA related MMP’s and especially C3M have shown to be increased with increasing fibrosis [24-28] and Ankylosing Spondylitis [29]. The formation markers are all measures of the N-terminal pro-collagen, which are also believed to be MMP derived. Moreover, to assess in more detail the degradation of cartilage and to control that the CIA model was working, the well-known CTX-II was used [30].

The aim of this study was to investigate the effect of autoimmunity by collagen induced arthritis (CIA) on type I, II, and III collagen turnover in rats utilizing the newly developed biomarker assays C1M, C2M, C3M, P1NP, P2NP, and P3NP, as well as CTX-II, before arthritis occur and at a late stage of arthritis.

## Methods

### Reagents

If not stated chemicals and reagents were purchased from Sigma-Aldrich (Copenhagen, Denmark) or VWR (Herlev, Denmark). The ELISA plate, pre-coated with streptavidin (nunc clear 96 well-plates), were purchased from Roche Diagnostics (cat.: 11940279, Mannheum, Germany). All ELISA plates were analyzed with the ELISA reader from Molecular Devices, SpectraMax M, (CA, USA). Rats were purchased from Charles River (Charles River, Germany). Antibodies were produced at Nordic Bioscience, Beijing. ELISA coating peptides was purchased from American Peptide Company.

### Collagen induced arthritis in rats

CIA was induced in 10, 7 week old, female Lewis rats by immunizing with 450 μl 2 mg/ml porcine type II collagen dissolved in 0.05 M acetic acid and emulsified 1:1 in Incomplete Freunds Adjuvant on day 0 and 7, as described earlier [30]. 10 Lewis rats only injected with 0.05 M acetic acid was used as control. Every day, starting from day 8, rats were examined for visual signs of disease, defined as macroscopic evidence of increase in paw size, determined paw score. A score from 0–4 was given depending on the swelling; 0: No evidence of erythema and swelling, 1: Erythema and mild swelling confined to the tarsals or ankle joint, 2: Erythema and mild swelling extending from the ankle to the tarsals, 3: Erythema and moderate swelling extending from the ankle to the metatarsal joints, 4: Erythema and severe swelling encompass the ankle, foot, and digits or ankylosis of the limb. Rats reaching a paw score of 10 were sacrificed, due to too great pain. Moreover, paw volume was measured. Paw volume is a widely used method to detect disease in the CIA model [31,32]. Paw volume increases due to inflammation, which is accompanied by interstitial oedema. We used a simple and accurate method to determine paw volume described before [32], with minor changes. Briefly, a 200 ml beaker with Mill-Q water was placed on a sensitive digital weight (sensitivity: 0.01 g). The rat’s paws were, one by one, lowered into the water. The sum of difference in weight from baseline for the paws was determined as the arbitrary number of paw size. The animal experiment protocol was approved by the local animal Ethics committee at Nordic Bioscience Beijing. The ethical approval number is NBB-AM-R/2009-01.

On day 16 all CIA rats were diseased, determined by paw score. The rats were sacrificed on day 26, besides if there were extensive paw swelling, determined by paw score, or a too great weight loss (>10%), then rats was terminated earlier. Serum samples were collected throughout the experiment from overnight fasted animals.

### Quantification of collagens

#### Degradation of type I collagen

The newly developed C1M ELISA detects degraded type I collagen by MMP-2, -9, and −13 [20]. The ELISA is based a monoclonal antibody (mAb) recognising the fragment GSPGKDGVRG at position 764 in the mature type I collagen. Briefly, 100 μl biotinylated coater peptide was added to a streptavitin coated plate. This was incubated at 20°C for 30 min, with agitation. Next, the plate was washed 5 times in washing buffer. Twenty μl sample or standard together with 100 μl HRP-labelled antibody was added to the plate. This was incubated at 20°C for 1 hour with agitation, followed by washing 5 times in washing buffer. One-hundred μl 3,3’,5,5’-tetramethylbenzidine (TMB) was added and incubated for 15 min. in the dark at 20°C with agitation. Lastly, 100 μl stop buffer (0.1% H_2_SO_4_) was added, before reading the plate at 540/590 nm on an ELISA reader, SpectraMax M. Technical data on C1M is listed in Table 1.

**Table 1:** Overview of the technical specifications of the ELISA’s applied in the experiment with CIA rats

**Assay name**	**Target**	**Specificity of assay**	**Detection range (ng/ml)**	**Intra-assay variation (%)**	**Inter-assay variation (%)**
C1M [20]	Degradation of type I collagen	Neo-epitope generated by MMP-2, 9, and 13	0.83-500	6.7	10.1
C2M [21]	Degradation of type II collagen	Neo-epitope generated by several MMP	0.09-2	3.5	7.3
C3M [22]	Degradation of type III collagen	Neo-epitope generated by MMP-9	0.86-50	2.7	3.4
P1NP [23]	Formation of type I collagen	N-terminal pro-peptide	0-1000	n.a.	n.a.
P2NP (unpublished)	Formation of type II collagen	N-terminal pro-peptide	0-1000	n.a.	n.a.
P3NP (unpublished)	Formation of type III collagen	N-terminal pro-peptide	0.149-76.3	4.1	11.0

#### Formation of type I collagen

The newly, previously described P1NP ELISA detects formation of type I collagen [23]. The assay is specific of a fragment (PEEYVSPDAEVIG) in the N-terminal pro-peptide of type I collagen. Briefly, 100 μl biotinylated coater peptide was added to a streptavidin coated plate and was incubated at 4°C for 30 min, with agitation. Next, the plate was washed 5 times in washing buffer. Twenty μl sample or standard together with 100 μl HRP-labelled antibody was added to the plate. This was incubated at 4°C for 1 hour with agitation, followed by washing 5 times in washing buffer. One-hundred μl TMB was added and incubated for 15 min. in the dark at 20°C with agitation. Lastly, 100 μl stop buffer (0.1% H_2_SO_4_) was added, before reading the plate at 540/590 nm on an ELISA reader, SpectraMax M. Technical data on P1NP is listed in Table 1.

#### Degradation of type II collagen

The novel, previously described, C2M (aka. CIIM) ELISA [21] detects a MMP-degradation fragment (RDGAAG) at position 1053 in the mature type II collagen. Moreover, the commercial available ELISA, CTX-II, detects degradation of type II collagen. This assay was run accordingly to the manufactures protocol. The protocol for C2M was briefly; 100 μl biotinylated coater peptide was added to a streptavidin coated plate and was incubated at 20°C for 30 min, with agitation. Next, the plate was washed 5 times in washing buffer. Twenty μl sample or standard together with 100 μl HRP-labelled antibody was added to the plate. This was incubated at 4°C for overnight with agitation, followed by washing 5 times in washing buffer. One-hundred μl TMB was added and incubated for 15 min. in the dark at 20°C with agitation. Lastly, 100 μl stop buffer (0.1% H_2_SO_4_) was added, before reading the plate at 540/590 nm on an ELISA reader, SpectraMax M. Technical data on C2M is listed in Table 1.

#### Formation of type II collagen

The novel P2NP ELISA detects the N-terminal pro-peptide of type II collagen (unpublished). A mAb was raised against the neo-epitope and an ELISA was made with the neo-epitope (biotinylated) as coater in an ELISA plate coated with streptavidin. The neo-epitope was used as standard [1000 ng/ml-0 ng/ml], to get a quantitative ELISA. Briefly, 100 μl biotinylated coater peptide was added to a streptavidin coated plate and was incubated at 20°C for 30 min, with agitation. Next, the plate was washed 5 times in washing buffer. Twenty μl sample or standard together with 100 μl HRP-labelled antibody was added to the plate. This was incubated at 4°C overnight with agitation, followed by washing 5 times in washing buffer. One-hundred μl TMB was added and incubated for 15 min. in the dark at 20°C with agitation. Lastly, 100 μl stop buffer (0.1% H_2_SO_4_) was added, before reading the plate at 540/590 nm on an ELISA reader, SpectraMax M. Technical data on P2NP is listed in Table 1.

#### Degradation of type III collagen

The C3M ELISA is a newly developed ELISA, which is specific of a MMP-9 degradation fragment (GGPGQPGTEGNK) of mature type III collagen [22]. Shortly, 100 μl biotinylated coater peptide was added to a streptavidin coated plate and was incubated at 20°C for 30 min, with agitation. Next, the plate was washed 5 times in washing buffer. Twenty μl sample or standard together with 100 μl HRP-labelled antibody was added to the plate. This was incubated at 20°C for 1 hour with agitation, followed by washing 5 times in washing buffer. One-hundred μl TMB was added and incubated for 15 min. in the dark at 20°C with agitation. Lastly, 100 μl stop buffer (0.1% H_2_SO_4_) was added, before reading the plate at 540/590 nm on an ELISA reader, SpectraMax M. Technical data on C3M is listed in Table 1.

#### Formation of type III collagen

The formation of type III collagen was quantified with the newly developed competitive P3NP assay (unpublished). The assay is specific of the fragment (PTGPQNYSP), which is a fragment in the N-terminal pro-peptide of type III collagen. A mAb was raised against the fragment and an ELISA was developed with the fragment (biotinylated) as coater in an ELISA plate coated with streptavidin. To quantify the formation level, the peptide was used a standard in the range [1000 ng/ml-0 ng/ml]. Briefly, 100 μl biotinylated coater peptide was added to a streptavidin coated plate and was incubated at 20°C for 30 min, with agitation. Next, the plate was washed 5 times in washing buffer. Twenty μl sample or standard together with 100 μl HRP-labelled antibody was added to the plate. This was incubated at 4°C overnight with agitation, followed by washing 5 times in washing buffer. One-hundred μl TMB was added and incubated for 15 min. in the dark at 20°C with agitation. Lastly, 100 μl stop buffer (0.1% H_2_SO_4_) was added, before reading the plate at 540/590 nm on an ELISA reader, SpectraMax M. Technical data on P3NP is listed in Table 1.

### Statistics

All values are presented as mean ± standard error of mean (SEM) with 10 replications in each of the groups. Significant difference between mean (day 7 and 22) and the day of first immunization (baseline) was determined using the non-parametric Mann–Whitney *t*-test. Differences were considered statistically significant if P < 0.05 and significant level as: * = P < 0.05, ** = P < 0.01, and ***:P < 0.001. The turnover of collagen was calculated by taken the ratio of formation compared to degradation.

## Results

### Animal health and disease incidence

**Figure 1:**
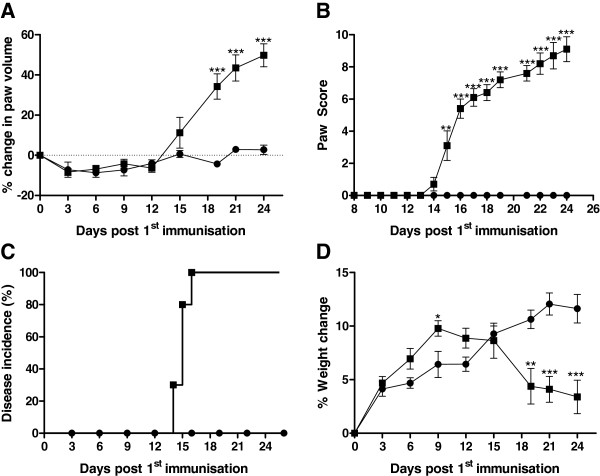
**The percentage increase in paw volume (A), paw score (B), percentage disease incidence (C), and percent weight changes (D) observed post 1^st^ immunization.** The CIA group (squares, n = 10) and control animals (circles, n = 10) were compared by Mann–Whitney *t*-test. All statistical analyses were calculated based on baseline corrected values. Data are shown as mean ± SEM and significance levels as *: P < 0.05, **: P < 0.01, ***: P < 0.001.

The earliest signs of disease in the CIA group were observed on day 14, measured by increase in paw volume (Figure 1A). By day 16 all immunized rats showed sign of disease (Figure 1C) and this time point was defined as disease onset. Paw volume increased in the diseased rat throughout the experiment, whereas no increase in paw volume was detected in the control group (Figure 1A). In addition, paw score was assessed and a significant difference between the groups was detected from day 15 and until termination of the experiment (Figure 1B). Three animals in the diseased group were sacrificed before the end of the study, due to a paw score higher than 10. The swelling of the paws was detected in the hind paws one to two days before signs of swelling were detected in the front paws. At the late stage, the activity of diseased rats were affected by the swelling, as most of the time, they were not active.

Figure 1D depicts the weight of the animals. The mean weight of the control group followed the normal weight gain for growing rats, with an increase of approximately 12% during the 3 weeks of the experiment. The mean weight of the CIA rat increased more rapidly until disease onset (day 16), whereafter the weight decreased significantly (p < 0.001) compared to the control group.

### Tissue balance of connective tissue, cartilage, and synovial membrane

**Figure 2:**
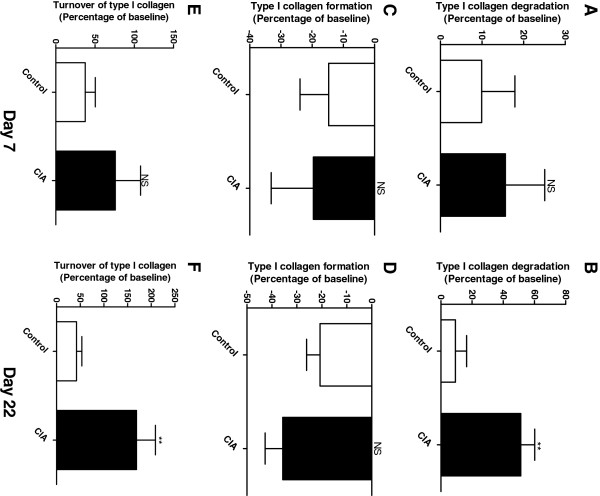
**Biomarkers of type I collagen turnover on day 7 and 22 corrected to baseline. A**, **B**: Type I collagen degradation detected with C1M. Range of measurement was [55.4-116.7]. **C**, **D**: Formation of type I collagen detected with P1NP. Range of measurement was [398.0-8603.1]. **E**, **F**: Calculations of type I collagen turnover. Range of measurement was [0.009-0.1]. The CIA group (black, n = 10) and control animals (white, n = 10) were compared by Mann–Whitney *t*-test. All statistical analysis was calculated based on baseline corrected values. Data are shown as mean ± SEM and significance levels as *: P < 0.05, **: P < 0.01, ***: P < 0.001.

The tissue balance of connective tissue was assessed by measurements of degradation and formation of type I collagen. The level of all biochemical markers of collagen is listed in Table 2. A 51.2% (P = 0.0068) increase in type I collagen degradation (C1M) was detected in serum from diseased animals on a late time point (day 22) compared to baseline level (Figure 2B). No difference in degradation of type I collagen was found between the groups before disease onset (day 7, Figure 2A). A decrease in formation of type I collagen (P1NP) compared to baseline level were detected in both treated and control animals during the experiment, but with no significance between the groups (Figure 2C, D). This imbalanced amount of degradation and formation, led to an overall significant increase in turnover (i.e. ratio between degradation and formation) of type I collagen on day 22 of 169% (P = 0.0052) in the diseased animals (Figure 2F). No significant difference was detected at the early time point in the control or diseased group (Figure 2E).

**Table 2:** Biochemical markers measured in serum on day 7 and 22 compared to baseline

	**Time point**	**Control**	**CIA**	**P-value**
C1M	Day 7	9.939 ± 7.963	15.63 ± 9.461	0.6038
	Day 22	9.283 ± 7.216	51.2 ± 8.947	0.0068 **
C2M	Day 7	5.376 ± 10.15	12.39 ± 11.54	0.5490
	Day 22	−7.593 ± 7.610	31.92 ± 10.51	0.0068 **
CTX-II	Day 7	−38.89 ± 41.61	−81.54 ± 6.355	0.7487
	Day 22	−87.71 ± 12.29	10963 ± 5236	0.0004 ***
C3M	Day 7	−1.609 ± 4.95	4.312 ± 8.117	0.6608
	Day 22	0.4491 ± 3.92	39.73 ± 5.447	<0.0001 ***
P1NP	Day 7	−14.72 ± 9.157	−19.59 ± 13.53	0.4002
	Day 22	−20.67 ± 5.433	,35.59 ± 7.097	0.2176
P2NP	Day 7	2.327 ± 13.29	−4.001 ± 5.772	0.8633
	Day 22	3.346 ± 11.57	−4.766 ± 9.643	0.8534
P3NP	Day 7	−1.829 ± 3.588	−8.746 ± 2.936	0.3562
	Day 22	2.334 ± 2.849	6.045 ± 2.790	0.5288

The CIA (n = 10) and control groups (n = 10) were compared by Man-Whitney t-test on both days. All statistical analyses were calculated based on baseline corrected values. Data are shown as mean ± SEM and significance levels as *: P < 0.05, **: P < 0.01, ***: P < 0.001.

**Figure 3:**
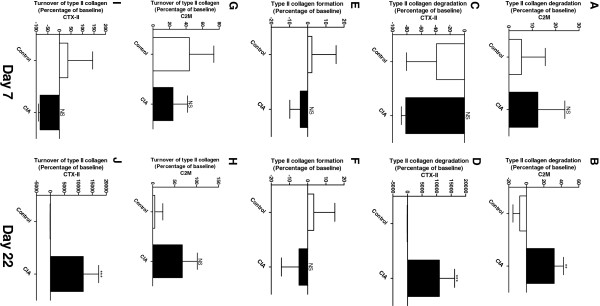
**Biomarkers of type II collagen turnover on day 7 and 22 corrected to baseline. A**, **B**: Type II collagen degradation detected with C2M. Range of measurement was [0.3-1.2]. **C**, **D**: Type II degradation detected with CTX-II. Range of measurement was [0.2-2132.7]. **E** + **F**. Formation of type II collagen detected with P2NP. Range of measurement was [167.3-877.5]. **G**, **H**: Calculations of type II collagen turnover with C2M. Range of measurement was [0.0005-0.006]. I + J: Calculation of type II collagen turnover with CTX-II. Range of measurement was [0.3-72.0]. The CIA group (black, n = 10) and control animals (white, n = 10) were compared by Mann Whitney *t*-test. All statistical analyses were calculated based on baseline corrected values. Data are shown as mean ± SEM and significance levels as *: P < 0.05, **: P < 0.01, ***: P < 0.001.

Type II collagen was used to determine the tissue balance of cartilage, as type II collagen is the main collagen of cartilage and it is not abundant in any other tissue. Degradation of type II collagen was measured by two different assays, utilizing two different neo-epitopes; the novel C2M ELISA [21] and the well-known CTX-II ELISA [33]. Both C2M and CTX-II showed significantly increased degradation in the diseased animals compared to control at the late time point, 31.9% (P = 0.0068) and 10963% (P = 0.0004), respectively (Figure 3B, D). However, CTX-II already showed increased degradation by more than 100% at disease onset in the treated animals compare to control (data not shown). No change in type II collagen degradation was detected before disease onset (Figure 3A, C). Furthermore, there was no significant difference between the formation of type II collagen (P2NP) in diseased animals compared to baseline or control (Figure 3E, F). Only at the late time point a significant increase (11765%, P = 0.0004) of turnover of type II collagen was detected with CTX-II (Figure 3J).

**Figure 4:**
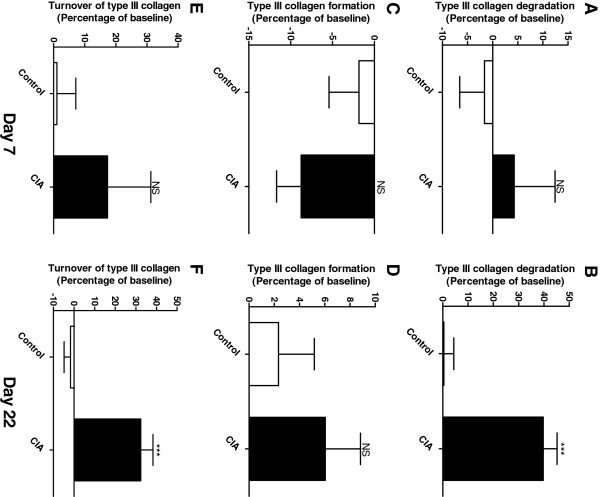
**Biomarkers of type III collagen turnover on day 7 and 22 corrected to baseline. A**, **B**: Type III collagen degradation detected with C3M. Range of measurement was [2.6-35.5]. **C**, **D**: Formation of type III collagen detected with P3NP. Range of measurement was [13.9-71.5]. **E**, **F**: Calculations of type III collagen turnover Range of measurement was [0.1-0.6]. The CIA group (black, n = 10) and control animals (white, n = 10) were compared by Mann-Whitneyt-test. All statistical analyses were calculated based on baseline corrected values. Data are shown as mean ± SEM and significance levels as *: P < 0.05, **: P < 0.01, ***: P < 0.001.

The tissue balance of connective tissue, including the synovial membrane, was determined by quantification of degradation and formation of type III collagen, the main collagen in the synovial membrane. The degradation of type III collagen (C3M) was increased with 39.7% (P < 0.0001) at the late time point in the diseased group compared to control (Figure 4B), but no change was detected at the early time point (Figure 4A). There was no significant difference in formation of type III collagen (P3NP) between the groups at the early or late time point (Figure 4C, D). However, a tendency towards elevated formation was detected in the diseased animals compared to baseline on the late time point. Overall, an elevated, but not significant, amount (17.4%, P = 0.24) of type III collagen turnover was detected in the diseased rats at the early time point (Figure 4E) and a significant increase in turnover was detected at the late time point (32.4%, P < 0.0001, Figure 4F).

## Discussion

Synovitis is the main characteristic of RA, however, the effect on the tissues involved of the increasing amount of proteolytic enzymes released into the synovial fluid due to the increase in synovial cells have not been studied in detail. Here we have used a CIA rat model to study the release of protein fragments of collagens, derived from cleavage with proteolytic enzymes, specifically MMP’s. An increase of degradation of type I, II, and III collagens was found, reflecting an elevated level of turnover of the collagens after disease onset. No significant difference in the formation of the collagens was detected between controls and diseased rats at any time point. In summary, the tissue balance of cartilage, synovial membrane, and connective tissue was disturbed.

The ELISA used to quantify type I collagen MMP-driven turnover (C1M [20], P1NP [23]) is not bone specific as conventional ELISAs of type I collagen, but the neo-epitope of the ELISA derives from connective tissues. C1M have earlier been found to significantly increase in ankylosing spondylitis patients compared to gender and age matched controls [29]. As there was no significant difference in the formation of type I collagen between the groups or compared to baseline in our study, an overall increase in type I collagen turnover was observed. This indicates an altered balance of connective tissue in the CIA rats towards degradation. Type I collagen is the most abundant collagen in vertebrates and is found in scar tissue, skeleton, synovial membrane, skin, tendons, ligaments, artery, etc. Many macromolecules, such as integrins, fibronectin, and decorin bind to type I collagen and certain cells like fibroblast attach to the ECM protein [34-36]. Therefore, the increased degradation of type I collagen in this experiment could reflect many metabolic processes. It could be that the skeleton and tendons weakens, due to decreased movement because of pain, or the increase in fibroblast in the synovial membrane increase the level of MMP’s increasing the degradation of type I collagen in the surrounding bone and synovial membrane. What exactly the degradation of type I collagen reflects in this experiment is still to be determined.

Type II collagen is the most abundant collagen in cartilage and CTX-II have in many studies been used to quantify cartilage degradation [30,37,38]. CTX-II has been shown to increase in RA patients receiving treatment (Cyclosporine, Methotrexate, and/or Steroid) compared to baseline. However, not even after four years of treatment a positive change in cartilage formation was detected, meaning that cartilage balance is compromised even at diagnosis of RA [37]. In the same study Christensen et al. showed that the CTX-II level was correlated to RA activity, demonstrating that the CTX-II level is coupled to synovitis activity [37]. In another study, a reduction in CTX-II level was found after three months in RA patients receiving treatment (prednisolone, Methotrexate, sulfasalazine alone or in combination) [39]. This result conflict with the earlier described study by Christensen et al. [37], where no positive effect of treatment was detected on cartilage. Together, these results could indicate that the right treatment could help the right patient group. However, there is no evidence for this theory yet.

A 250% increase in cartilage degradation quantified by CTX-II in serum was detected at a late time point in diseased rats in a pre-clinical study of CIA rats. However, an even greater increase (400%) in degradation was detected between the diseased rats and controls, when quantifying CTX-II in paw extracts [30]. However, alone CTX-II only reflects a part of the mechanisms of cartilage breakdown, suggesting that a panel of biomarkers of type II collagen degradation are to be used to get a broader understanding of mechanisms of cartilage breakdown. In this study we used both C2M and CTX-II, which showed relatively different levels of degradation, however the same profile. Both markers of type II collagen was used, not only to get a broader understanding of collagen breakdown, but also because CTX-II was used a control of the CIA model working, as CTX-II have been described in the model previously [30]. C2M is detecting a neo-epitope of type II collagen derived from MMP-cleavage, which is specific of articular cartilage [21], where the neo-epitope in CTX-II is also derived from MMP-cleavage, but it is present during both bone sclerosis and growth plate remodelling [40]. In this experiment the CTX-II fragment was detected to a much higher extend (343 times) than the C2M fragment. This may partly reflect the release of several different MMPs from synovial fibroblasts in the inflamed synovial membrane, which could account for the difference in degradation level of cartilage reflected in this study. A difference in cartilage breakdown, when comparing two biomarkers, has previously been shown with CTX-II and Helix-II [41]. In this study Charni-Ben et al. suggested using various biomarkers reflecting the same metabolic process concurrently to increase the knowledge about the metabolic process. This is true in the case of basal science, but when applied to clinical science, measuring several biomarkers reflecting one metabolic process will be overestimating. In the clinic, a panel of biomarkers reflecting different metabolisms in a given disease gives a broader understanding of the specific case and a better tool to diagnose. Moreover, as both biomarkers of cartilage degradation showed the same profile of cartilage breakdown in our experiment, one biomarker of cartilage degradation is adequate to estimate the state of cartilage balance. As CTX-II is only detectable in human urine and not human serum and C2M is detectable in both human urine and serum, C2M is a better candidate for use in the clinics, as blood samples are already used to detect C-Reactive Protein and Rheumatoid factor and urine samples will therefore not be needed.

The compromised balance of connective tissue and cartilage turnover is, as stated earlier, a result of the thickened synovial membrane. As RA is an inflammatory disease of the synovial joint, a prognostic RA biomarker panel must include biomarkers to detect the state of the inflamed synovial membrane. This could potentially be used to detect RA in an early stage, i.e. before joint degradation. In this study, we used the turnover of type III collagen, as a marker of the synovial membrane. As the synovial membrane thickness, due to fibrogenesis, an increase in P3NP and no change in C3M were expected. However, a significant (P > 0.0001) increase in degradation of type III collagen was detected at the late time point, but no significant difference in formation was found between CIA and control rats in this experiment, though a tendency towards increased P3NP in diseased rats was detected at the late time point. This suggests inhibited fibrogenesis in the diseased rats after disease onset and therefore not an increase of synovial membrane thickness. However, the N-terminal propeptide of type III collagen, on which P3NP is based, is not always cleaved of before fibrillation [42]. Therefore, this quantification of formation of type III collagen may be an underestimation and misleading. Moreover, it is very likely that C3M derives from other tissues and not exclusively from the synovial membrane. As the synovial joints swells great quickly in the CIA model, an increase in type III collagen formation (P3NP) was expected, which would correlate to synovial membrane thickness. However, this seems not to be the case.

Type III collagen degradation and formation separately have in several studies been used to detect fibrogenesis [24-28], however this study is the first to combine the biomarkers and investigate tissue balance. In three different fibrosis models (two of liver and one of skin), type III collagen degradation was measured with the C3M ELISA assay. A significantly increase in C3M was detected even after a short disease period in rats compared to control rats [24-26]. However, P3NP is also increased in fibrosis, as the amount of tissue is increased. Myllylä et al. [43] found that P3NP was increased in RA patients compared to controls and Hakala et al. [44] even showed that P3NP was increased in RA patients compared to osteoarthritis, psoriasis arthritis, ankylosing spondylitis, and systemic lupus erythematosus patients. Other groups have further enhanced the knowledge that P3NP increases with fibrosis [27,28]. To our knowledge P3NP has not been measured along with degradation markers of type III collagen in any murine or human trials, thereby investigating fibrogenesis. We show that fibrogenesis of the synovial membrane is inhibited in CIA rats. As especially C3M has shown to be a possible diagnostic marker of fibrogenesis in rat [24-26] and in Ankylosing Spondylitis patients [29], an investigation if C3M could be an efficacy marker for RA could be interesting. We propose to treat CIA animals with MTX or another RA related drug, to investigate if the level of C3M decreases with treatment. Type III collagen degradation will by itself not be specific of RA, as this has also shown to be elevated in ankylosing spondylitis patients. However, the right combination of collagen biomarkers could be RA specific.

The diagnosis of RA is today based on imaging, self-reported symptoms, and biochemical markers. However, all of these biomarkers and symptoms are only applicable on late stage disease. Therefore, a biomarker panel of early diagnosis of RA is warranted, as this will increase the chances of treatment, maybe even cure. It has been showed that cartilage collagen regeneration is compromised at a very early stage of RA and that this is not primarily inflammation driven [37], increasing the evidence that articular cartilage degradation is a possible early RA biomarker. CTX-II could be one of these early non-invasive biomarkers as CTX-II has been shown to predict joint damage at 4 and 12 weeks in human trials [38]. However, as C2M is an articular cartilage specific biochemical marker, C2M is a better candidate than CTX-II in an early non-invasive biomarker panel of RA, but more studies confirming it as an early marker is needed. In a measurement of multiple surface proteins (a biomarker panel), Fueldner et al. showed a better discrimination of RA and controls, than when only determining one surface protein [45]. This further increases the evidence of a biomarker panel to be better diagnostic or prognostic than single biomarkers [46]. Moreover, as the pathology of RA involved several cell types, it is reasonable to detect the activation state of more than one cell type.

In this experiment the weight of the rats in the CIA groups was significantly increased before disease onset (day 9). This could be due to water accumulation in the tissues. After disease onset the weight of the diseased rats decrease compared to control rats. This weight loss at the late time point could be because of a decrease in bone and muscle mass, due to reduced activity in the diseased rats. As three rats were sacrificed before experiment termination and the decreased activity of the rats, we propose to use another model of RA, to increase consistency and animal number at experiment termination.

## Conclusions

In summary, we found increased turnover of type I, II, and III collagen, indicating an altered balance of these collagens in RA. The profile of the tissue balances indicates that cartilage, synovial membrane, and connective tissue are important events in RA. The novel C3M biomarker could discriminate between diseased and controls, indicating this as a promising biomarker of synovitis. We speculate that a combination of these biological relevant collagen turnover markers might be utilised in a biomarker panel as diagnostic and prognostic markers of RA.

## Abbreviations

C1M: MMP degraded type I collagen; C2M: MMP degraded type II collagen; C3M: MMP degraded type III collagen; CIA: Collagen induced arthritis; CTX-II: C-terminal crosslinked telopeptide type II collagen; ELISA: Enzyme-linked immunosorbent assay; P1NP: N-terminal neo-epitope of type I procollagen; P2NP: N-terminal neo-epitope of type II procollagen; P3NP: N-terminal neo-epitope of type III procollagen; RA: Rheumatoid arthritis; TBM: 3,3’,5,5-tetramethylbenzidine.

## Competing interests

Morten Karsdal is stock holder of Nordic Bioscience.

## Authors’ contributions

ASS and AC B-J have designed the experiment. JW, JY and ZQ performed the animal experiment. ASS measured the biochemical markers and did the data analysis. Lastly AC B-J and MK did proofreading. All authors read and approved the final manuscript prior to submission and re-submission.
